# Supplementary material for: Environmental DNA metabarcoding of water samples as a tool for monitoring Iberian freshwater fish composition
Source: PLoS One. 2023 Oct 30;18(10):e0283088. doi: 10.1371/journal.pone.0283088 (PMC10615313; doi:10.1371/journal.pone.0283088)
Supplement: S1 File — (DOCX) [file pone.0283088.s001.docx]

**Supplementary information for:**

**Environmental DNA metabarcoding of water samples as a tool for monitoring Iberian freshwater fish composition**

Andrea Corral-Lou^1,2*^ and Ignacio Doadrio^1^

^1^Biodiversity and Evolutionary Biology Department, Museo Nacional de Ciencias Naturales, CSIC, José Gutiérrez Abascal, 2; 28006 Madrid, Spain

^2^Consultores en Biología de la Conservación S.L., Daoiz, 12; 28004 Madrid, Spain

*** Corresponding author**

**E-mail:** ancoralou@gmail.com (ACL)

**ORCID:** 0000-0003-0895-1305

**Authors' Contributions**

ID designed the research and revised the manuscript. ACL collected data, made the analyses and wrote the manuscript.

**Table S1.** Species used in the present study to expand the 12S reference database. The table shows both the species obtained from Genbank (G) and the species sequenced in the present study (P). It also includes the total number of haplotypes obtained for each species (H) and the total number of individuals analyzed for each species (N). The accession number of Genbank of the sequences obtained in this study is marked in bold.

| **Species** | **G** | **P** | **H** | **N** | **Accession number of Genbank sequences** |
| --- | --- | --- | --- | --- | --- |
| *A. arcasii* | 0 | 8 | 3 | 8 | **OP739007-OP739014** |
| *A. salmantinum* | 0 | 5 | 2 | 5 | **OP739058-OP739062** |
| *A. alburnus* | 2 | 1 | 1 | 3 | AB239593; NC_008659; **OP739006** |
| *A. melas* | 1 | 0 | 1 | 1 | KT804702.1 |
| *B. barbatula* | 2 | 2 | 2 | 4 | KP715096; NC027192; **OP739017-OP739018** |
| *C. auratus* | 44 | 2 | 3 | 46 | AB111951; GU086395; GU086396; GU086397; JN105355; AB379915; AB379916; AB379917; AB379918; AB379919; AB379920; AB379921; AP011236; KF147851; KJ476998; KJ874428; KJ874429; KJ874430; KJ874431; KM657133; KM659025; KT756205; KU146528; KX505165; KY798482; MF443758; MF443759; MF443760; MF443761; MF443762; MF443763; MF443764; MF443765; MF443766; MF443767; MF443768; MF443769; MF443770; MF443771; HQ875340; KM657134; NC_015142; AP011239; KM657132; **OP739015-OP739016** |
| *C. calderoni* | 0 | 4 | 2 | 4 | **OP739028-OP739031** |
| *C. paludica* | 0 | 4 | 1 | 4 | **OP739054-OP739057** |
| *C. vettonica* | 0 | 5 | 2 | 5 | **OP739071-OP739075** |
| *C. carpio* | 41 | 2 | 4 | 43 | AP009047; MK134852; AP017364; AP017365; JN105352; JN105353; JN105354; JN105357; JX188253; KF856964; KF856965; KF932266; KJ511882; KJ511883; KP013086; KP993136; KP993137; KP993138; KP993139; KU050703; KU146529; KU146530; KU159761; KU301745; KX710076; KY798484; MG570426; MG570427; MG570435; MH202953; MK088487; MK291479; MT780875; NC_018035; NC_018036; NC_018037; NC_018039; NC_018366; JX188254; AP017363; NC_001606; **OP739038-OP739039** |
| *E. lucius* | 2 | 0 | 1 | 2 | AP004103; NC_004593 |
| *G. holbrooki* | 4 | 1 | 1 | 5 | KP013085; NC_028274; KP013115; KY798480; **OP739047** |
| *G. lozanoi* | 0 | 6 | 1 | 6 | **OP739048-OP739053** |
| *L. aspius* | 1 | 0 | 1 | 1 | MK574834.2 |
| *L. gibbosus* | 5 | 2 | 2 | 7 | KP013097; NC_028284; MF621724; MF621725; MF621726; **OP739045-OP739046** |
| *L. bocagei* | 0 | 6 | 1 | 6 | **OP739022-OP739027** |
| *L. rifensis* | 1 | 0 | 1 | 1 | KX348041.1 |
| *M. salmoides* | 8 | 0 | 3 | 8 | NC_014686; MH301076; MH301075; MH301074; HQ391896; NC_008106; DQ536425; AP014537 |
| *O. mykiss* | 17 | 0 | 5 | 17 | AF113120; AF113121; AF125508; AF125509; L29771; NC_001717; CF752467; HQ167664; DQ288269; DQ288270; DQ288271; KP013084; KY798500; LC050735; DQ288268; KP085590; MF621750 |
| *P. bigerri* | 0 | 3 | 1 | 3 | **OP739019-OP739021** |
| *P. duriense* | 0 | 5 | 1 | 5 | **OP739040-OP739044** |
| *P. polylepis* | 2 | 0 | 1 | 2 | AP011282.1; NC_031574.1 |
| *S. trutta* | 16 | 6 | 2 | 22 | AM910409; HQ167666; JQ390057; KT633607; KY798501; LT617630; LT617631; LT617632; MF621760; MF621761; MF621762; MF621763; NC_010007; NC_024032; GU233801; KT634053; **OP739065-OP739070** |
| *S. alburnoides* | 0 | 8 | 2 | 8 | **OP738998-OP739005** |
| *S. carolitertti* | 0 | 6 | 1 | 6 | **OP739032-OP739037** |
| *T. tinca* | 3 | 2 | 1 | 5 | AB218686; KY798486; NC_008648; **OP739063-OP739064** |
| **Total** | 149 | 78 | 46 | 227 |  |

**Tabla S2.** Intraspecific and interspecific percentage of similarity for the *12S* gene. Allochthonous species are marked with an asterisk.

|  | *A.alburnus_1* | *L.aspius_1* | *A.arcasiicea_1* | *A.arcasiidur_1* | *A.salmantinum_1* | *A.salmantinum_2* | *P.duriense_1* | *P.polylepis_1* | *A.arcasiicorn_1* | *S.alburnoides_1* | *S.alburnoides_2* | *S.carolitertii_1* | *P.bigerri_1* | *G.lozanoi_1* | *T.tinca_1* | *C.auratus_1* | *C.auratus_2* | *C.auratus_3* | *C.carpio_1* | *C.carpio_2* | *C.carpio_3* | *C.carpio_4* | *L.bocagei_1* |
| --- | --- | --- | --- | --- | --- | --- | --- | --- | --- | --- | --- | --- | --- | --- | --- | --- | --- | --- | --- | --- | --- | --- | --- |
| *A.alburnus_1** |  |  |  |  |  |  |  |  |  |  |  |  |  |  |  |  |  |  |  |  |  |  |  |
| *L.aspius_1** | 97.7 |  |  |  |  |  |  |  |  |  |  |  |  |  |  |  |  |  |  |  |  |  |  |
| *A.arcasiicea_1* | 92.6 | 92.6 |  |  |  |  |  |  |  |  |  |  |  |  |  |  |  |  |  |  |  |  |  |
| *A.arcasiidur_1* | 93.1 | 93.1 | 96 |  |  |  |  |  |  |  |  |  |  |  |  |  |  |  |  |  |  |  |  |
| *A.salmantinum_1* | 95.4 | 95.4 | 97.1 | 97.7 |  |  |  |  |  |  |  |  |  |  |  |  |  |  |  |  |  |  |  |
| *A.salmantinum_2* | 94.9 | 94.9 | 96.6 | 97.1 | 99.4 |  |  |  |  |  |  |  |  |  |  |  |  |  |  |  |  |  |  |
| *P.duriense_1* | 94.3 | 94.3 | 97.1 | 95.4 | 97.7 | 97.1 |  |  |  |  |  |  |  |  |  |  |  |  |  |  |  |  |  |
| *P.polylepis_1* | 93.7 | 93.7 | 96.6 | 94.9 | 97.1 | 96.6 | 99.4 |  |  |  |  |  |  |  |  |  |  |  |  |  |  |  |  |
| *A.arcasiicorn_1* | 93.7 | 93.7 | 94.3 | 97.1 | 97.1 | 96.6 | 96 | 95.4 |  |  |  |  |  |  |  |  |  |  |  |  |  |  |  |
| *S.alburnoides_1* | 94.3 | 94.3 | 94.9 | 93.1 | 95.4 | 94.9 | 96.6 | 96 | 93.7 |  |  |  |  |  |  |  |  |  |  |  |  |  |  |
| *S.alburnoides_2* | 94.3 | 94.3 | 94.9 | 93.1 | 95.4 | 94.9 | 95.4 | 94.9 | 93.7 | 98.9 |  |  |  |  |  |  |  |  |  |  |  |  |  |
| *S.carolitertii_1* | 94.9 | 94.9 | 94.3 | 93.7 | 96 | 95.4 | 96 | 96.6 | 94.3 | 98.3 | 98.3 |  |  |  |  |  |  |  |  |  |  |  |  |
| *P.bigerri_1** | 88.6 | 88.6 | 90.3 | 88.6 | 89.7 | 89.1 | 90.3 | 89.7 | 88.6 | 92.6 | 91.4 | 90.9 |  |  |  |  |  |  |  |  |  |  |  |
| *G.lozanoi_1* | 89.3 | 89.3 | 87 | 88.7 | 89.8 | 89.3 | 88.1 | 87.6 | 89.8 | 90.4 | 90.4 | 91 | 88.1 |  |  |  |  |  |  |  |  |  |  |
| *T.tinca_1* | 88.7 | 89.8 | 88.7 | 88.1 | 90.4 | 89.8 | 90.4 | 89.8 | 89.8 | 89.8 | 89.8 | 90.4 | 88.1 | 91.5 |  |  |  |  |  |  |  |  |  |
| *C.auratus_1** | 85.8 | 88.1 | 83.5 | 84.7 | 85.2 | 84.7 | 84.7 | 84.1 | 86.4 | 85.8 | 85.8 | 85.8 | 83 | 88.7 | 88.7 |  |  |  |  |  |  |  |  |
| *C.auratus_2** | 85.2 | 87.5 | 84.1 | 84.1 | 84.7 | 84.1 | 84.1 | 83.5 | 85.8 | 85.2 | 86.4 | 85.2 | 82.4 | 88.1 | 88.1 | 99.4 |  |  |  |  |  |  |  |
| *C.auratus_3** | 86.9 | 89.2 | 84.7 | 84.7 | 86.4 | 85.8 | 85.8 | 85.2 | 86.4 | 86.9 | 86.9 | 86.9 | 84.1 | 88.7 | 89.8 | 98.8 | 98.3 |  |  |  |  |  |  |
| *C.carpio_1** | 84.1 | 86.4 | 84.1 | 85.2 | 85.8 | 85.2 | 84.1 | 83.5 | 85.8 | 85.2 | 85.2 | 85.2 | 83.5 | 89.3 | 87.6 | 97.1 | 96.5 | 96.5 |  |  |  |  |  |
| *C.carpio_2** | 84.7 | 86.9 | 84.7 | 85.8 | 86.4 | 85.8 | 84.7 | 84.1 | 86.4 | 85.8 | 85.8 | 85.8 | 83.5 | 89.3 | 88.7 | 98.3 | 97.7 | 97.7 | 98.8 |  |  |  |  |
| *C.carpio_3** | 84.1 | 86.4 | 84.7 | 85.8 | 86.4 | 85.8 | 84.7 | 84.1 | 86.4 | 85.2 | 85.2 | 85.2 | 83 | 88.7 | 89.3 | 97.7 | 97.1 | 97.1 | 98.3 | 99.4 |  |  |  |
| *C.carpio_4** | 84.7 | 86.9 | 84.7 | 85.8 | 86.4 | 85.8 | 84.7 | 84.1 | 86.4 | 84.7 | 84.7 | 84.7 | 83.5 | 88.1 | 89.8 | 97.1 | 96.5 | 96.5 | 97.7 | 98.8 | 98.3 |  |  |
| *L.bocagei_1* | 86.9 | 88.1 | 86.4 | 86.4 | 86.9 | 86.4 | 88.1 | 87.5 | 88.1 | 88.6 | 89.2 | 88.6 | 85.8 | 91 | 90.4 | 93.7 | 93.7 | 93.7 | 93.7 | 93.7 | 93.1 | 92.5 |  |
| *L.rifensis_1* | 86.9 | 88.1 | 86.4 | 86.4 | 86.9 | 86.4 | 88.1 | 87.5 | 88.1 | 88.6 | 89.2 | 88.6 | 86.9 | 89.8 | 89.3 | 93.1 | 93.1 | 93.1 | 93.1 | 93.1 | 92.5 | 92 | 98.3 |
| *B.barbatula_1* | 81.7 | 82.9 | 81.7 | 81.7 | 84 | 83.4 | 83.4 | 82.9 | 82.9 | 81.1 | 81.1 | 81.1 | 78.3 | 80.8 | 81.9 | 81.6 | 81 | 82.8 | 82.2 | 82.8 | 82.8 | 82.8 | 83.4 |
| *B.barbatula_2* | 82.3 | 83.4 | 82.3 | 82.3 | 84.6 | 84 | 84 | 83.4 | 83.4 | 81.7 | 81.7 | 81.7 | 78.9 | 81.4 | 82.5 | 81.6 | 81 | 82.8 | 82.2 | 82.8 | 82.8 | 82.8 | 83.4 |
| *C.calderoni_1* | 84.1 | 85.2 | 83.5 | 83 | 84.7 | 84.1 | 84.7 | 84.1 | 83.5 | 85.8 | 85.2 | 85.2 | 82.4 | 83.1 | 82.6 | 84.1 | 83.5 | 85.2 | 83.5 | 84.1 | 83.5 | 83 | 84.7 |
| *C.calderoni_2* | 84.1 | 85.2 | 83.5 | 83 | 84.7 | 84.1 | 84.7 | 84.1 | 83.5 | 85.8 | 85.2 | 85.2 | 82.4 | 83.1 | 82.6 | 84.1 | 83.5 | 85.2 | 83.5 | 84.1 | 83.5 | 83 | 84.7 |
| *C.paludica_1* | 82.4 | 83.5 | 82.4 | 82.4 | 83.5 | 83 | 83 | 82.4 | 82.4 | 84.1 | 83.5 | 83.5 | 81.8 | 82.6 | 80.9 | 83.4 | 82.9 | 84 | 83.4 | 84 | 83.4 | 82.9 | 84.1 |
| *C.vettonica_1* | 81.8 | 83 | 81.8 | 81.8 | 83 | 82.4 | 82.4 | 81.8 | 81.8 | 83.5 | 83 | 83 | 82.4 | 83.1 | 81.5 | 83.4 | 82.9 | 84 | 83.4 | 84 | 83.4 | 82.9 | 83.5 |
| *C.vettonica_2* | 81.8 | 83 | 81.8 | 81.8 | 83 | 82.4 | 82.4 | 81.8 | 81.8 | 83.5 | 83 | 83 | 82.4 | 83.1 | 81.5 | 83.4 | 82.9 | 84 | 83.4 | 84 | 83.4 | 82.9 | 83.5 |
| *A.melas_1** | 74.4 | 75.6 | 75 | 76.1 | 76.7 | 76.1 | 75.6 | 75 | 77.3 | 77.3 | 77.3 | 77.3 | 75.6 | 79.2 | 78.7 | 81 | 80.5 | 80.5 | 81.6 | 81.6 | 82.2 | 81.6 | 78.9 |
| *E.lucius_1** | 68.6 | 69.1 | 67.4 | 67.4 | 68.6 | 68 | 68 | 67.4 | 68 | 68.6 | 69.1 | 68.6 | 69.1 | 66.7 | 67.8 | 67.6 | 67.6 | 67.6 | 67.6 | 67.1 | 67.6 | 66.5 | 68.4 |
| *O.mykiss_1** | 73.1 | 73.7 | 73.1 | 72.6 | 74.3 | 74.9 | 73.7 | 73.1 | 74.3 | 74.9 | 75.4 | 75.4 | 74.3 | 74.6 | 75.7 | 74.7 | 74.7 | 74.7 | 77 | 75.9 | 75.3 | 75.9 | 76 |
| *O.mykiss_3** | 72.2 | 72.7 | 72.2 | 71.6 | 73.3 | 73.9 | 72.7 | 72.2 | 73.3 | 73.9 | 74.4 | 74.4 | 73.3 | 73.6 | 74.7 | 73.7 | 73.7 | 73.7 | 76 | 74.9 | 74.3 | 74.9 | 75 |
| *O.mykiss_4** | 72.7 | 73.3 | 72.7 | 72.2 | 73.9 | 74.4 | 73.3 | 72.7 | 73.9 | 74.4 | 75 | 75 | 73.9 | 74.2 | 75.3 | 74.3 | 74.3 | 74.3 | 76.6 | 75.4 | 74.9 | 75.4 | 75.6 |
| *O.mykiss_5** | 72.2 | 72.7 | 72.2 | 71.6 | 73.3 | 73.9 | 72.7 | 72.2 | 73.3 | 73.9 | 74.4 | 74.4 | 73.3 | 73.6 | 74.7 | 73.7 | 73.7 | 73.7 | 76 | 74.9 | 74.3 | 74.9 | 75 |
| *O.mykiss_2** | 72.2 | 72.7 | 72.2 | 71.6 | 73.3 | 73.9 | 72.7 | 72.2 | 73.3 | 73.9 | 74.4 | 74.4 | 73.3 | 73.6 | 74.7 | 73.7 | 73.7 | 73.7 | 76 | 74.9 | 74.3 | 74.9 | 75 |
| *S.trutta_1* | 71 | 71.6 | 68.8 | 69.9 | 71 | 71.6 | 69.3 | 68.8 | 71.6 | 70.5 | 71 | 71 | 70.5 | 72.5 | 71.3 | 72 | 72 | 72 | 73.7 | 72.6 | 72 | 72.6 | 72.7 |
| *S.trutta_2* | 70.5 | 71 | 69.3 | 70.5 | 71.6 | 72.2 | 69.9 | 69.3 | 72.2 | 71 | 71.6 | 71.6 | 70.5 | 71.9 | 71.9 | 71.4 | 71.4 | 71.4 | 73.1 | 72 | 71.4 | 72 | 72.2 |
| *L.gibbosus_1** | 71.4 | 72 | 69.7 | 69.7 | 70.9 | 71.4 | 69.7 | 69.1 | 70.9 | 71.4 | 72.6 | 71.4 | 70.3 | 71.8 | 68.9 | 73 | 73.6 | 73 | 71.8 | 73 | 72.4 | 71.8 | 69.7 |
| *L.gibbosus_2** | 72 | 72.6 | 70.3 | 70.3 | 71.4 | 72 | 70.3 | 69.7 | 71.4 | 72 | 73.1 | 72 | 70.9 | 71.8 | 68.9 | 73 | 73.6 | 73 | 71.8 | 73 | 72.4 | 71.8 | 69.7 |
| *M.salmoides_1** | 70.9 | 71.4 | 69.7 | 70.3 | 71.4 | 70.9 | 70.9 | 70.3 | 72 | 72 | 72.6 | 72 | 69.1 | 70.6 | 70.6 | 71.3 | 71.3 | 71.3 | 71.3 | 71.3 | 71.8 | 70.1 | 70.9 |
| *M.salmoides_2** | 71.4 | 72 | 70.3 | 70.9 | 72 | 71.4 | 71.4 | 70.9 | 72.6 | 72.6 | 73.1 | 72.6 | 69.7 | 71.2 | 71.2 | 71.8 | 71.8 | 71.8 | 71.8 | 71.8 | 72.4 | 70.7 | 71.4 |
| *M.salmoides_3** | 71.4 | 72 | 70.3 | 70.9 | 72 | 71.4 | 71.4 | 70.9 | 72.6 | 72.6 | 73.1 | 72.6 | 69.7 | 71.8 | 70.6 | 71.3 | 71.3 | 71.3 | 72.4 | 71.3 | 71.8 | 70.1 | 72 |
| *G.holbrooki_1** | 66.5 | 67.6 | 68.8 | 67 | 67.6 | 67 | 67 | 66.5 | 65.9 | 68.8 | 68.2 | 67.6 | 67.6 | 67.4 | 67.4 | 67.4 | 67.4 | 67.4 | 68.6 | 69.1 | 68.6 | 68.6 | 67.6 |

|  | *L.rifensis_1* | *B.barbatula_1* | *B.barbatula_2* | *C.calderoni_1* | *C.calderoni_2* | *C.paludica_1* | *C.vettonica_1* | *C.vettonica_2* | *A.melas_1* | *E.lucius_1* | *O.mykiss_1* | *O.mykiss_3* | *O.mykiss_4* | *O.mykiss_5* | *O.mykiss_2* | *S.trutta_1* | *S.trutta_2* | *L.gibbosus_1* | *L.gibbosus_2* | *M.salmoides_1* | *M.salmoides_2* | *M.salmoides_3* |
| --- | --- | --- | --- | --- | --- | --- | --- | --- | --- | --- | --- | --- | --- | --- | --- | --- | --- | --- | --- | --- | --- | --- |
| *B.barbatula_1* | 82.3 |  |  |  |  |  |  |  |  |  |  |  |  |  |  |  |  |  |  |  |  |  |
| *B.barbatula_2* | 82.3 | 99.4 |  |  |  |  |  |  |  |  |  |  |  |  |  |  |  |  |  |  |  |  |
| *C.calderoni_1* | 83.6 | 85.1 | 85.6 |  |  |  |  |  |  |  |  |  |  |  |  |  |  |  |  |  |  |  |
| *C.calderoni_2* | 83.6 | 85.1 | 85.6 | 99.4 |  |  |  |  |  |  |  |  |  |  |  |  |  |  |  |  |  |  |
| *C.paludica_1* | 83 | 85.5 | 86.1 | 96.6 | 96.6 |  |  |  |  |  |  |  |  |  |  |  |  |  |  |  |  |  |
| *C.vettonica_1* | 82.4 | 84.4 | 85 | 97.1 | 97.1 | 98.3 |  |  |  |  |  |  |  |  |  |  |  |  |  |  |  |  |
| *C.vettonica_2* | 82.4 | 84.4 | 85 | 96 | 96 | 98.3 | 98.8 |  |  |  |  |  |  |  |  |  |  |  |  |  |  |  |
| *A.melas_1** | 78.3 | 79.2 | 79.2 | 77.1 | 77.1 | 78.7 | 78.2 | 78.2 |  |  |  |  |  |  |  |  |  |  |  |  |  |  |
| *E.lucius_1** | 69 | 66.5 | 66.5 | 66.9 | 66.9 | 67.2 | 67.2 | 66.7 | 69.4 |  |  |  |  |  |  |  |  |  |  |  |  |  |
| *O.mykiss_1** | 76.6 | 73.4 | 73.4 | 73.7 | 73.7 | 74.1 | 74.7 | 73.6 | 77.6 | 81.1 |  |  |  |  |  |  |  |  |  |  |  |  |
| *O.mykiss_3** | 75.6 | 72.4 | 72.4 | 72.7 | 72.7 | 73.1 | 73.7 | 72.6 | 77.7 | 80 | 98.8 |  |  |  |  |  |  |  |  |  |  |  |
| *O.mykiss_4** | 76.1 | 73 | 73 | 73.3 | 73.3 | 73.7 | 74.3 | 73.1 | 77.1 | 80.6 | 99.4 | 99.4 |  |  |  |  |  |  |  |  |  |  |
| *O.mykiss_5** | 75.6 | 72.4 | 72.4 | 72.7 | 72.7 | 73.1 | 73.7 | 72.6 | 76.6 | 80 | 98.8 | 98.8 | 99.4 |  |  |  |  |  |  |  |  |  |
| *O.mykiss_2** | 75.6 | 72.4 | 72.4 | 72.7 | 72.7 | 73.1 | 73.7 | 72.6 | 77.7 | 80 | 98.8 | 98.8 | 98.2 | 97.7 |  |  |  |  |  |  |  |  |
| *S.trutta_1* | 73.3 | 70.7 | 70.7 | 71 | 71 | 71.4 | 72 | 70.9 | 76 | 80 | 94.1 | 95.3 | 94.7 | 94.1 | 94.2 |  |  |  |  |  |  |  |
| *S.trutta_2* | 72.7 | 70.1 | 70.1 | 70.5 | 70.5 | 70.9 | 71.4 | 70.3 | 76 | 80 | 94.7 | 95.9 | 95.3 | 94.7 | 94.7 | 99.4 |  |  |  |  |  |  |
| *L.gibbosus_1** | 69.7 | 71.8 | 72.4 | 72 | 72 | 72 | 72.6 | 72 | 71.8 | 71.6 | 74.7 | 73.7 | 74.3 | 73.7 | 73.7 | 73.7 | 73.7 |  |  |  |  |  |
| *L.gibbosus_2** | 69.7 | 72.4 | 73 | 72.6 | 72.6 | 72.6 | 73.1 | 72.6 | 72.4 | 72.2 | 75.9 | 74.9 | 75.4 | 74.9 | 74.9 | 73.7 | 73.7 | 98.8 |  |  |  |  |
| *M.salmoides_1** | 70.9 | 73.6 | 74.1 | 73.7 | 73.7 | 72.6 | 73.1 | 72.6 | 74.1 | 75.7 | 75.9 | 74.9 | 75.4 | 74.9 | 74.9 | 74.9 | 74.9 | 85.8 | 85.8 |  |  |  |
| *M.salmoides_2** | 71.4 | 74.1 | 74.7 | 74.3 | 74.3 | 73.1 | 73.7 | 73.1 | 74.7 | 76.3 | 76.5 | 75.4 | 76 | 75.4 | 75.4 | 75.4 | 75.4 | 86.4 | 86.4 | 99.4 |  |  |
| *M.salmoides_3** | 72 | 74.1 | 74.7 | 74.3 | 74.3 | 73.1 | 73.7 | 73.1 | 73.6 | 77.5 | 77.1 | 76 | 76.6 | 76 | 76 | 76 | 76 | 85.2 | 85.2 | 98.2 | 98.8 |  |
| *G.holbrooki_1** | 66.5 | 67.4 | 68 | 69.3 | 69.3 | 68.2 | 68.8 | 68.2 | 69.1 | 67.4 | 67.6 | 67.8 | 67.2 | 66.7 | 67.8 | 67.8 | 67.2 | 73.8 | 72.7 | 73.3 | 73.8 | 73.8 |

**Table S3.** The relative abundance of obtained reads for each sample (BL, L1 and L2) and locality. The summer and winter samples are indicated as M1 and M2 respectively. The green color highlights cases where a species was only detected by one of the two liters.

| **M1** | **Arlanza**  **(1)** | | | **Pisuerga**  **(3)** | | **Boedo**  **(2)** | | | **Tera**  **(12)** | | | **Aliste**  **(6)** | | **Esla**  **(4)** | | **Órbigo**  **(5)** | | **Yeltes**  **(7)** | | **Corneja**  **(8)** | | **Voltoya**  **(11)** | | **Adaja**  **(9)** | | **Voltoya**  **(10)** | |
| --- | --- | --- | --- | --- | --- | --- | --- | --- | --- | --- | --- | --- | --- | --- | --- | --- | --- | --- | --- | --- | --- | --- | --- | --- | --- | --- | --- |
|  | BL | L1 | L2 | L1 | L2 | BL | L1 | L2 | BL | L1 | L2 | L1 | L2 | L1 | L2 | L1 | L2 | L1 | L2 | L1 | L2 | L1 | L2 | L1 | L2 | L1 | L2 |
| *Conger* |  |  |  |  |  |  |  |  |  | 0.21 |  |  |  |  |  |  |  |  |  |  |  |  |  |  |  |  |  |
| *T. japonicus* |  |  |  |  |  |  |  |  |  |  | 1.29 |  |  |  |  |  |  |  |  |  |  |  |  |  |  |  |  |
| *T. trachurus* |  |  |  |  |  |  |  |  |  | 0.19 | 0.09 |  |  |  |  |  |  |  |  |  |  |  |  | 0.09 | 0.12 |  |  |
| *L. gibbosus* |  | 1.12 | 0.52 |  |  |  |  |  |  |  |  | 73.93 | 72.36 | 0.25 | 0.48 |  | 0.05 | 5.42 | 28.09 |  |  |  |  |  |  |  |  |
| *S. pilchardus* |  |  |  |  |  |  |  |  |  |  |  |  |  |  |  |  |  |  |  |  |  |  |  | 1.79 | 1.73 |  |  |
| *C. calderoni* |  | 0.43 | 1.36 |  |  |  | 0.84 | 0.46 |  | 0.59 | 0.32 |  |  |  |  | 1.43 | 4.02 |  |  |  |  |  |  |  |  |  |  |
| *C. paludica* |  |  |  |  |  |  |  |  |  |  |  |  |  |  |  |  |  | 21.06 | 16.6 |  |  |  |  |  |  |  |  |
| *C. auratus* |  |  |  |  |  |  |  |  |  |  |  |  |  |  |  |  |  |  |  |  |  |  |  | 0.66 | 0.44 |  |  |
| *C. carpio* |  |  |  |  |  |  |  |  |  |  |  |  |  |  |  |  |  |  |  |  |  | 0.73 | 1.38 | 3.42 | 3.97 |  |  |
| *Cyprinus* |  |  |  |  |  |  |  |  |  |  |  |  |  |  |  |  |  |  |  |  |  |  |  | 0.19 | 0.22 |  |  |
| *L. bocagei* |  | 3.58 | 3.67 |  |  |  |  |  |  | 15.85 | 18.58 |  |  | 16.72 | 18.73 |  |  | 0.47 | 9.93 | 1.27 | 0.24 | 3.95 | 4.3 |  |  |  |  |
| *L. rifensis* |  | 0.07 |  |  |  |  |  |  |  |  |  |  |  |  |  |  |  |  |  |  |  |  |  |  |  |  |  |
| *Luciobarbus* |  | 0.07 | 0.11 |  |  |  |  |  |  | 0.5 | 0.54 |  |  | 0.7 | 0.33 |  |  |  | 0.13 |  |  |  |  |  |  |  |  |
| *G. lozanoi* |  | 25.08 | 29.74 | 3.42 | 2.9 |  | 32.48 | 32.74 |  | 29.67 | 31.34 | 6.62 | 8.75 | 39.16 | 38.88 | 0.49 | 1.79 |  |  |  |  |  |  | 65.37 | 64.22 | 80.26 | 82.58 |
| *Gobio* |  | 0.65 | 0.76 |  |  |  | 0.34 | 0.41 |  | 1.31 | 1.79 |  |  | 1.88 | 1.35 |  |  |  |  |  |  |  |  | 1.24 | 1.74 | 0.34 |  |
| *A. arcasii* (3) |  |  |  |  |  |  |  |  |  |  |  |  |  | 0.51 | 0.13 | 0.39 | 1.13 |  |  |  |  |  |  |  |  |  |  |
| *A. arcasii* (2) |  |  |  |  |  |  |  |  |  |  |  |  |  |  |  |  |  |  |  | 93.67 | 94.01 |  |  |  |  |  |  |
| *A. arcasii* (1) |  | 0.15 | 0.85 |  | 0.26 |  | 29.1 | 28.14 |  | 16.89 | 13.67 | 0.19 |  |  | 0.18 |  |  |  |  | 4.54 | 5.54 | 0.55 | 0.68 | 21.98 | 22.01 | 18.45 | 17.42 |
| *A. salmantinum* |  |  |  |  |  |  |  |  |  |  |  |  |  |  |  |  |  | 2.2 | 2.89 |  |  |  |  |  |  |  |  |
| *A. alburnus* |  |  |  |  |  |  |  |  |  |  |  | 18.08 | 17.56 | 8.27 | 8.56 |  |  | 0.66 | 1.07 |  |  |  |  |  |  |  |  |
| *Alburnus* |  |  |  |  |  |  |  |  |  |  |  |  |  | 0.43 |  |  |  |  |  |  |  |  |  |  |  |  |  |
| *Chondrostoma* |  | 0.03 | 0.14 |  |  |  | 0.38 | 0.34 |  | 0.52 | 0.61 |  |  |  |  |  |  |  |  | 0.13 |  |  |  | 1.32 | 1.15 | 0.4 |  |
| *Leuciscidae* |  |  |  |  |  |  |  |  |  | 0.15 | 0.19 |  |  |  |  |  |  |  |  |  |  |  |  | 0.11 | 0.09 |  |  |
| *Phoxinus* | 100 | 1.75 | 1.59 | 0.88 | 0.46 |  |  |  |  |  |  |  |  |  |  |  |  |  |  |  |  |  |  |  |  |  |  |
| *P. bigerri* |  | 58.89 | 50.39 | 68.76 | 66.59 |  |  |  |  |  |  |  |  |  | 0.1 |  |  |  |  |  |  |  |  |  |  |  |  |
| *P. duriense* |  | 3.54 | 5.15 | 14.45 | 16.05 |  |  |  |  |  |  |  |  | 0.27 | 0.29 |  |  | 3.12 | 7.88 | 0.17 | 0.21 |  |  |  |  |  |  |
| *P. polylepis* |  |  |  |  |  |  |  |  |  |  |  |  |  |  |  |  |  |  |  |  |  |  |  | 0.17 |  |  |  |
| *Squalius* |  | 0.08 | 0.08 |  |  |  |  |  |  | 0.41 | 0.72 |  |  |  |  |  |  |  |  |  |  |  |  | 0.35 | 0.18 |  |  |
| *S. alburnoides* |  |  |  |  |  |  |  |  |  |  |  |  |  |  |  |  |  |  |  | 0.22 |  |  |  |  |  |  |  |
| *S. carolitertii* |  | 3.06 | 2.19 |  |  |  |  |  |  | 9.22 | 15.21 |  |  |  | 0.28 |  |  | 3.52 | 21.21 |  |  |  |  | 2.93 | 2.5 |  |  |
| *B. barbatula* |  |  |  |  |  |  |  |  |  |  |  |  |  | 27.17 | 22.9 | 85.56 | 69.48 |  |  |  |  |  |  |  |  |  |  |
| *Barbatula* |  |  |  |  |  |  |  |  |  |  |  |  |  | 0.15 | 0.1 | 0.15 | 0.12 |  |  |  |  |  |  |  |  |  |  |
| *T. tinca* |  |  |  |  |  |  |  |  |  |  |  | 0.3 | 0.21 | 1.08 | 4.2 |  | 0.15 |  | 2.48 |  |  |  |  |  |  | 0.55 |  |
| *G. holbrooki* |  |  |  |  |  |  |  |  |  |  |  |  |  |  |  | 2.43 | 5.34 | 63.55 | 9.28 |  |  | 15.33 | 59.15 |  |  |  |  |
| *Gambusia* |  |  |  |  |  |  |  |  |  |  |  |  |  |  |  | 0.06 | 0.06 |  |  |  |  | 0.16 | 0.12 |  |  |  |  |
| *E. lucius* |  |  |  |  |  |  |  |  |  |  |  | 0.47 | 1.12 | 0.23 | 0.2 |  |  |  |  |  |  |  |  |  |  |  |  |
| *D. labrax* |  |  |  |  |  |  |  |  |  |  |  |  |  | 0.55 | 1.08 |  | 0.12 |  |  |  |  |  |  |  |  |  |  |
| *S. maximus* |  |  |  |  |  |  |  |  |  |  |  |  |  |  |  |  |  |  |  |  |  |  |  | 0.08 |  |  |  |
| *O. mykiss* |  |  |  |  |  |  |  |  |  |  |  |  |  | 0.07 |  | 0.24 | 0.86 |  |  |  |  |  |  |  |  |  |  |
| *Oncorhynchus* |  |  |  |  |  |  |  |  |  |  |  |  |  |  |  | 0.12 | 0.19 |  |  |  |  |  |  |  |  |  |  |
| *Salmo* |  |  |  | 0.08 | 0.1 |  |  |  | 0.15 |  |  |  |  |  |  | 0.08 |  |  |  |  |  |  |  |  |  |  |  |
| *S. trutta* |  | 1.5 | 3.45 | 12.41 | 13.65 | 100 | 36.86 | 37.9 | 99.85 | 24.49 | 15.57 | 0.42 |  | 2.56 | 2.2 | 9.04 | 16.7 |  | 0.44 |  |  | 0.1 |  |  |  |  |  |
| *T. alalunga* |  |  |  |  |  |  |  |  |  |  |  |  |  |  |  |  |  |  |  |  |  |  |  |  | 0.09 |  |  |
| *A. melas* |  |  |  |  |  |  |  |  |  |  |  |  |  |  |  |  |  |  |  |  |  | 79.06 | 34.12 |  |  |  |  |
| *Ameiurus* |  |  |  |  |  |  |  |  |  |  |  |  |  |  |  |  |  |  |  |  |  | 0.13 | 0.24 |  |  |  |  |
| *S. aurata* |  |  |  |  |  |  |  |  |  |  | 0.08 |  |  |  |  |  |  |  |  |  |  |  |  | 0.29 | 1.53 |  |  |

| **M2** | **Esla**  **(4)** | | **Órbigo**  **(5)** | | **Adaja**  **(9)** | | **Tera**  **(12)** | |
| --- | --- | --- | --- | --- | --- | --- | --- | --- |
|  | L1 | L2 | L1 | L2 | L1 | L2 | L1 | L2 |
| *C. calderoni* |  |  | 1.59 | 1.35 |  |  |  |  |
| *L. bocagei* | 8.79 | 6.69 |  |  |  |  | 0.32 | 0.54 |
| *Luciobarbus* | 0.17 | 0.08 |  |  |  |  |  |  |
| *G. lozanoi* | 31.73 | 38.44 | 1.17 | 2.15 | 49.98 | 47.93 | 39.99 | 40.9 |
| *Gobio* | 0.74 | 0.79 |  |  | 0.99 | 1.18 | 0.9 | 0.79 |
| *A. arcasii* (3) | 3.98 | 3.77 | 1.1 | 1.16 |  |  |  |  |
| *A. arcasii* (1) |  |  |  |  | 46.16 | 48.33 | 20.95 | 23.13 |
| *Achondrostoma* | 0.16 |  |  |  | 1.06 | 0.93 | 0.79 | 0.7 |
| *A. alburnus* | 12.42 | 10.87 |  |  |  |  |  | 0.08 |
| *Alburnus* | 0.35 | 0.32 |  |  |  |  |  |  |
| *Leuciscus* | 0.16 | 0.21 |  |  |  |  |  |  |
| *Leuciscidae* |  |  |  |  | 0.06 |  | 0.17 | 0.19 |
| *Pseudochondrostoma* |  | 0.15 |  |  |  |  |  |  |
| *P. duriense* | 8.66 | 8.53 |  |  |  |  | 0.2 |  |
| *P. polylepis* |  |  |  |  | 0.08 | 0.13 |  |  |
| *Squalius* |  |  |  |  | 0.07 |  | 0.58 | 0.52 |
| *S. carolitertii* |  | 0.39 |  | 0.06 | 1.6 | 1.5 | 8.58 | 7.99 |
| *B. barbatula* | 16.92 | 15.61 | 27.88 | 27.68 |  |  |  |  |
| *G. holbrooki* |  |  | 8.09 | 12.84 |  |  |  |  |
| *E. lucius* | 0.46 | 0.76 |  | 0.11 |  |  |  |  |
| *D. labrax* | 0.17 | 0.09 |  |  |  |  |  |  |
| *Hippoglossus* |  |  | 1.16 | 1.91 |  |  |  |  |
| *L. aspera* |  |  | 0.07 |  |  |  |  |  |
| *Salmonidae* |  |  | 0.06 | 0.11 |  |  |  |  |
| *O. mykiss* | 3.51 | 4.5 | 3.53 | 2.56 |  |  |  |  |
| *Oncorhynchus* | 1.62 | 2.14 | 1.39 | 1.5 |  |  |  |  |
| *Salmo* | 0.43 | 0.2 | 0.6 | 0.84 |  |  | 0.09 | 0.08 |
| *S. trutta* | 9.7 | 6.47 | 53.36 | 47.75 |  |  | 27.43 | 25.08 |

**Table S4.** The number of assigned reads for each sample (BL, L1 and L2), locality and species. The summer and fall samples are indicated as M1 and M2 respectively.

| **ID M1**  **Species** | AT5138_BL | AT5138_L1 | AT5138_L2 | AT5140_L1 | AT5140_L2 | AT5141_BL | AT5141_L1 | AT5141_L2 | AT5169_BL | AT5169_L1 | AT5169_L2 | AT5143_L1 | AT5143_L2 | AT5144_L1 | AT5144_L2 | AT5145_L1 | AT5145_L2 | AT5152_L1 | AT5152_L2 | AT5153_L1 | AT5153_L2 | AT5154_L1 | AT5154_L2 | AT5155_L1 | AT5155_L2 | AT5156_L1 | AT5156_L2 |
| --- | --- | --- | --- | --- | --- | --- | --- | --- | --- | --- | --- | --- | --- | --- | --- | --- | --- | --- | --- | --- | --- | --- | --- | --- | --- | --- | --- |
| *Conger* | 0 | 0 | 0 | 0 | 0 | 0 | 0 | 0 | 0 | 32 | 0 | 0 | 0 | 0 | 0 | 0 | 0 | 0 | 0 | 0 | 0 | 0 | 0 | 0 | 0 | 0 | 0 |
| *T. japonicus* | 0 | 0 | 0 | 0 | 0 | 0 | 0 | 0 | 0 | 0 | 222 | 0 | 0 | 0 | 0 | 0 | 0 | 0 | 0 | 0 | 0 | 0 | 0 | 0 | 0 | 0 | 0 |
| *T. trachurus* | 0 | 0 | 0 | 0 | 0 | 0 | 0 | 0 | 0 | 28 | 15 | 0 | 0 | 0 | 0 | 0 | 0 | 0 | 0 | 0 | 0 | 0 | 0 | 15 | 22 | 0 | 0 |
| *L. gibbosus* | 0 | 372 | 148 | 0 | 0 | 0 | 0 | 0 | 0 | 0 | 0 | 5876 | 4573 | 45 | 57 | 0 | 11 | 498 | 3992 | 0 | 0 | 0 | 0 | 0 | 0 | 0 | 0 |
| *S. pilchardus* | 0 | 0 | 0 | 0 | 0 | 0 | 0 | 0 | 0 | 0 | 0 | 0 | 0 | 0 | 0 | 0 | 0 | 0 | 0 | 0 | 0 | 0 | 0 | 288 | 321 | 0 | 0 |
| *C. calderoni* | 0 | 143 | 386 | 0 | 0 | 0 | 115 | 62 | 0 | 89 | 55 | 0 | 0 | 0 | 0 | 401 | 851 | 0 | 0 | 0 | 0 | 0 | 0 | 0 | 0 | 0 | 0 |
| *C. paludica* | 0 | 0 | 0 | 0 | 0 | 0 | 0 | 0 | 0 | 0 | 0 | 0 | 0 | 0 | 0 | 0 | 0 | 1933 | 2359 | 0 | 0 | 0 | 0 | 0 | 0 | 0 | 0 |
| *C. auratus* | 0 | 0 | 0 | 0 | 0 | 0 | 0 | 0 | 0 | 0 | 0 | 0 | 0 | 0 | 0 | 0 | 0 | 0 | 0 | 0 | 0 | 0 | 0 | 107 | 81 | 0 | 0 |
| *C. carpio* | 0 | 0 | 0 | 0 | 0 | 0 | 0 | 0 | 0 | 0 | 0 | 0 | 0 | 0 | 0 | 0 | 0 | 0 | 0 | 0 | 0 | 217 | 443 | 550 | 737 | 0 | 0 |
| *Cyprinus* | 0 | 0 | 0 | 0 | 0 | 0 | 0 | 0 | 0 | 0 | 0 | 0 | 0 | 0 | 0 | 0 | 0 | 0 | 0 | 0 | 0 | 0 | 0 | 31 | 41 | 0 | 0 |
| *L. bocagei* | 0 | 1191 | 1046 | 0 | 0 | 0 | 0 | 0 | 0 | 2389 | 3209 | 0 | 0 | 2964 | 2239 | 0 | 0 | 43 | 1411 | 106 | 17 | 1182 | 1383 | 0 | 0 | 0 | 0 |
| *L. rifensis* | 0 | 23 | 0 | 0 | 0 | 0 | 0 | 0 | 0 | 0 | 0 | 0 | 0 | 0 | 0 | 0 | 0 | 0 | 0 | 0 | 0 | 0 | 0 | 0 | 0 | 0 | 0 |
| *Luciobarbus* | 0 | 23 | 30 | 0 | 0 | 0 | 0 | 0 | 0 | 76 | 94 | 0 | 0 | 124 | 40 | 0 | 0 | 0 | 19 | 0 | 0 | 0 | 0 | 0 | 0 | 0 | 0 |
| *G. lozanoi* | 0 | 8336 | 8466 | 592 | 573 | 0 | 4452 | 4367 | 0 | 4471 | 5413 | 526 | 553 | 6941 | 4649 | 138 | 378 | 0 | 0 | 0 | 0 | 0 | 0 | 10520 | 11909 | 2627 | 2409 |
| *Gobio* | 0 | 217 | 217 | 0 | 0 | 0 | 47 | 55 | 0 | 198 | 310 | 0 | 0 | 333 | 162 | 0 | 0 | 0 | 0 | 0 | 0 | 0 | 0 | 200 | 323 | 11 | 0 |
| *A. arcasiicea (3)* | 0 | 0 | 0 | 0 | 0 | 0 | 0 | 0 | 0 | 0 | 0 | 0 | 0 | 91 | 15 | 110 | 239 | 0 | 0 | 0 | 0 | 0 | 0 | 0 | 0 | 0 | 0 |
| *A. arcasii (2)* | 0 | 0 | 0 | 0 | 0 | 0 | 0 | 0 | 0 | 0 | 0 | 0 | 0 | 0 | 0 | 0 | 0 | 0 | 0 | 7817 | 6633 | 0 | 0 | 0 | 0 | 0 | 0 |
| *A. arcasii (1)* | 0 | 49 | 243 | 0 | 52 | 0 | 3989 | 3754 | 0 | 2545 | 2361 | 15 | 0 | 0 | 22 | 0 | 0 | 0 | 0 | 379 | 391 | 163 | 220 | 3538 | 4082 | 604 | 508 |
| *A. salmantinum* | 0 | 0 | 0 | 0 | 0 | 0 | 0 | 0 | 0 | 0 | 0 | 0 | 0 | 0 | 0 | 0 | 0 | 202 | 411 | 0 | 0 | 0 | 0 | 0 | 0 | 0 | 0 |
| *A. alburnus* | 0 | 0 | 0 | 0 | 0 | 0 | 0 | 0 | 0 | 0 | 0 | 1437 | 1110 | 1465 | 1024 | 0 | 0 | 61 | 152 | 0 | 0 | 0 | 0 | 0 | 0 | 0 | 0 |
| *Alburnus* | 0 | 0 | 0 | 0 | 0 | 0 | 0 | 0 | 0 | 0 | 0 | 0 | 0 | 76 | 0 | 0 | 0 | 0 | 0 | 0 | 0 | 0 | 0 | 0 | 0 | 0 | 0 |
| *Chondrostoma* | 0 | 11 | 40 | 0 | 0 | 0 | 52 | 46 | 0 | 78 | 105 | 0 | 0 | 0 | 0 | 0 | 0 | 0 | 0 | 11 | 0 | 0 | 0 | 212 | 213 | 13 | 0 |
| *Leuciscidae* | 0 | 0 | 0 | 0 | 0 | 0 | 0 | 0 | 0 | 23 | 33 | 0 | 0 | 0 | 0 | 0 | 0 | 0 | 0 | 0 | 0 | 0 | 0 | 18 | 17 | 0 | 0 |
| *Phoxinus* | 31321 | 583 | 453 | 153 | 91 | 0 | 0 | 0 | 0 | 0 | 0 | 0 | 0 | 0 | 0 | 0 | 0 | 0 | 0 | 0 | 0 | 0 | 0 | 0 | 0 | 0 | 0 |
| *P. bigerri* | 0 | 19574 | 14345 | 11903 | 13166 | 0 | 0 | 0 | 0 | 0 | 0 | 0 | 0 | 0 | 12 | 0 | 0 | 0 | 0 | 0 | 0 | 0 | 0 | 0 | 0 | 0 | 0 |
| *P. duriense* | 0 | 1178 | 1467 | 2502 | 3173 | 0 | 0 | 0 | 0 | 0 | 0 | 0 | 0 | 47 | 35 | 0 | 0 | 286 | 1120 | 14 | 15 | 0 | 0 | 0 | 0 | 0 | 0 |
| *P. polylepis* | 0 | 0 | 0 | 0 | 0 | 0 | 0 | 0 | 0 | 0 | 0 | 0 | 0 | 0 | 0 | 0 | 0 | 0 | 0 | 0 | 0 | 0 | 0 | 28 | 0 | 0 | 0 |
| *Squalius* | 0 | 25 | 23 | 0 | 0 | 0 | 0 | 0 | 0 | 62 | 125 | 0 | 0 | 0 | 0 | 0 | 0 | 0 | 0 | 0 | 0 | 0 | 0 | 56 | 34 | 0 | 0 |
| *S. alburnoides* | 0 | 0 | 0 | 0 | 0 | 0 | 0 | 0 | 0 | 0 | 0 | 0 | 0 | 0 | 0 | 0 | 0 | 0 | 0 | 18 | 0 | 0 | 0 | 0 | 0 | 0 | 0 |
| *S. carolitertii* | 0 | 1016 | 623 | 0 | 0 | 0 | 0 | 0 | 0 | 1389 | 2627 | 0 | 0 | 0 | 33 | 0 | 0 | 323 | 3014 | 0 | 0 | 0 | 0 | 471 | 463 | 0 | 0 |
| *B. barbatula* | 0 | 0 | 0 | 0 | 0 | 0 | 0 | 0 | 0 | 0 | 0 | 0 | 0 | 4815 | 2738 | 24027 | 14711 | 0 | 0 | 0 | 0 | 0 | 0 | 0 | 0 | 0 | 0 |
| *Barbatula* | 0 | 0 | 0 | 0 | 0 | 0 | 0 | 0 | 0 | 0 | 0 | 0 | 0 | 27 | 12 | 41 | 25 | 0 | 0 | 0 | 0 | 0 | 0 | 0 | 0 | 0 | 0 |
| *T. tinca* | 0 | 0 | 0 | 0 | 0 | 0 | 0 | 0 | 0 | 0 | 0 | 24 | 13 | 192 | 502 | 0 | 31 | 0 | 352 | 0 | 0 | 0 | 0 | 0 | 0 | 18 | 0 |
| *G. holbrooki* | 0 | 0 | 0 | 0 | 0 | 0 | 0 | 0 | 0 | 0 | 0 | 0 | 0 | 0 | 0 | 683 | 1131 | 5834 | 1318 | 0 | 0 | 4583 | 19005 | 0 | 0 | 0 | 0 |
| *Gambusia* | 0 | 0 | 0 | 0 | 0 | 0 | 0 | 0 | 0 | 0 | 0 | 0 | 0 | 0 | 0 | 18 | 12 | 0 | 0 | 0 | 0 | 49 | 38 | 0 | 0 | 0 | 0 |
| *E. lucius* | 0 | 0 | 0 | 0 | 0 | 0 | 0 | 0 | 0 | 0 | 0 | 37 | 71 | 40 | 24 | 0 | 0 | 0 | 0 | 0 | 0 | 0 | 0 | 0 | 0 | 0 | 0 |
| *D. labrax* | 0 | 0 | 0 | 0 | 0 | 0 | 0 | 0 | 0 | 0 | 0 | 0 | 0 | 98 | 129 | 0 | 25 | 0 | 0 | 0 | 0 | 0 | 0 | 0 | 0 | 0 | 0 |
| *S. maximus* | 0 | 0 | 0 | 0 | 0 | 0 | 0 | 0 | 0 | 0 | 0 | 0 | 0 | 0 | 0 | 0 | 0 | 0 | 0 | 0 | 0 | 0 | 0 | 13 | 0 | 0 | 0 |
| *O. mykiss* | 0 | 0 | 0 | 0 | 0 | 0 | 0 | 0 | 0 | 0 | 0 | 0 | 0 | 12 | 0 | 68 | 182 | 0 | 0 | 0 | 0 | 0 | 0 | 0 | 0 | 0 | 0 |
| *Oncorhynchus* | 0 | 0 | 0 | 0 | 0 | 0 | 0 | 0 | 0 | 0 | 0 | 0 | 0 | 0 | 0 | 33 | 41 | 0 | 0 | 0 | 0 | 0 | 0 | 0 | 0 | 0 | 0 |
| *Salmo* | 0 | 0 | 0 | 13 | 19 | 0 | 0 | 0 | 25 | 0 | 0 | 0 | 0 | 0 | 0 | 23 | 0 | 0 | 0 | 0 | 0 | 0 | 0 | 0 | 0 | 0 | 0 |
| *S. trutta* | 0 | 499 | 981 | 2149 | 2697 | 404 | 5054 | 5056 | 16501 | 3690 | 2690 | 33 | 0 | 453 | 263 | 2539 | 3537 | 0 | 62 | 0 | 0 | 29 | 0 | 0 | 0 | 0 | 0 |
| *T. alalunga* | 0 | 0 | 0 | 0 | 0 | 0 | 0 | 0 | 0 | 0 | 0 | 0 | 0 | 0 | 0 | 0 | 0 | 0 | 0 | 0 | 0 | 0 | 0 | 0 | 16 | 0 | 0 |
| *Ameiurus* | 0 | 0 | 0 | 0 | 0 | 0 | 0 | 0 | 0 | 0 | 0 | 0 | 0 | 0 | 0 | 0 | 0 | 0 | 0 | 0 | 0 | 40 | 76 | 0 | 0 | 0 | 0 |
| *S. aurata* | 0 | 0 | 0 | 0 | 0 | 0 | 0 | 0 | 0 | 0 | 14 | 0 | 0 | 0 | 0 | 0 | 0 | 0 | 0 | 0 | 0 | 0 | 0 | 47 | 284 | 0 | 0 |
| *A. melas* | 0 | 0 | 0 | 0 | 0 | 0 | 0 | 0 | 0 | 0 | 0 | 0 | 0 | 0 | 0 | 0 | 0 | 0 | 0 | 0 | 0 | 23638 | 10964 | 0 | 0 | 0 | 0 |

| **ID M2**  **Species** | AT5166_L1 | AT5166_L2 | AT5167_L1 | AT5167_L2 | AT5168_L1 | AT5168_L2 | AT5169_L1 | AT5169_L2 |
| --- | --- | --- | --- | --- | --- | --- | --- | --- |
| *C. calderoni* | 0 | 0 | 329 | 340 | 0 | 0 | 0 | 0 |
| *L. bocagei* | 607 | 1017 | 0 | 0 | 0 | 0 | 67 | 120 |
| *Luciobarbus* | 12 | 12 | 0 | 0 | 0 | 0 | 0 | 0 |
| *G. lozanoi* | 2190 | 5848 | 243 | 542 | 9537 | 6772 | 8246 | 9051 |
| *Gobio* | 51 | 120 | 0 | 0 | 189 | 167 | 185 | 175 |
| *A. arcasiicea* | 275 | 573 | 228 | 292 | 0 | 0 | 0 | 0 |
| *A. arcasiidur* | 0 | 0 | 0 | 0 | 8808 | 6829 | 4320 | 5118 |
| *Achondrostoma* | 11 | 0 | 0 | 0 | 202 | 131 | 162 | 154 |
| *A. alburnus* | 857 | 1654 | 0 | 0 | 0 | 0 | 0 | 17 |
| *Alburnus* | 24 | 48 | 0 | 0 | 0 | 0 | 0 | 0 |
| *Leuciscus* | 11 | 32 | 0 | 0 | 0 | 0 | 0 | 0 |
| *Leuciscidae* | 0 | 0 | 0 | 0 | 11 | 0 | 36 | 42 |
| *Pseudochondrostoma* | 0 | 23 | 0 | 0 | 0 | 0 | 0 | 0 |
| *P. duriense* | 598 | 1298 | 0 | 0 | 0 | 0 | 41 | 0 |
| *P. polylepis* | 0 | 0 | 0 | 0 | 16 | 18 | 0 | 0 |
| *Squalius* | 0 | 0 | 0 | 0 | 14 | 0 | 119 | 115 |
| *S. carolitertii* | 0 | 59 | 0 | 15 | 306 | 212 | 1770 | 1768 |
| *B. barbatula* | 1168 | 2374 | 5771 | 6973 | 0 | 0 | 0 | 0 |
| *G. holbrooki* | 0 | 0 | 1674 | 3235 | 0 | 0 | 0 | 0 |
| *E. lucius* | 32 | 116 | 0 | 28 | 0 | 0 | 0 | 0 |
| *D. labrax* | 12 | 13 | 0 | 0 | 0 | 0 | 0 | 0 |
| *Hippoglossus* | 0 | 0 | 241 | 480 | 0 | 0 | 0 | 0 |
| *L. aspera* | 0 | 0 | 15 | 0 | 0 | 0 | 0 | 0 |
| *Salmonidae* | 0 | 0 | 12 | 27 | 0 | 0 | 0 | 0 |
| *O. mykiss* | 242 | 685 | 731 | 644 | 0 | 0 | 0 | 0 |
| *Oncorhynchus* | 112 | 326 | 288 | 377 | 0 | 0 | 0 | 0 |
| *Salmo* | 30 | 30 | 124 | 210 | 0 | 0 | 19 | 17 |
| *S. trutta* | 670 | 985 | 11047 | 12024 | 0 | 0 | 5656 | 5552 |

**Table S5.** The number of unassigned reads for each sample (BL, L1 and L2), locality and species after remove reads with an alignment coverage with the matched sequence from GenBank lesser than 100% and/or the percentage of similarity lesser than 97%. The summer and fall samples are indicated as M1 and M2 respectively.

| **ID M1**  **Species** | AT5138_BL | AT5138_L1 | AT5138_L2 | AT5140_BL | AT5140_L1 | AT5140_L2 | AT5141_BL | AT5141_L1 | AT5141_L2 | AT5142_BL | AT5142_L1 | AT5142_L2 | AT5143_BL | AT5143_L1 | AT5143_L2 | AT5144_BL | AT5144_L1 | AT5144_L2 | AT5145_BL | AT5145_L1 | AT5145_L2 | AT5152_BL | AT5152_L1 | AT5152_L2 | AT5153_BL | AT5153_L1 | AT5153_L2 | AT5154_BL | AT5154_L1 | AT5154_L2 | AT5155_BL | AT5155_L1 | AT5155_L2 | AT5156_BL | AT5156_L1 | AT5156_L2 | **ATBPCRA** |
| --- | --- | --- | --- | --- | --- | --- | --- | --- | --- | --- | --- | --- | --- | --- | --- | --- | --- | --- | --- | --- | --- | --- | --- | --- | --- | --- | --- | --- | --- | --- | --- | --- | --- | --- | --- | --- | --- |
| **Troglodytes troglodytes (Aves)** | **0** | **0** | **0** | **0** | **0** | **0** | **0** | **0** | **0** | **0** | **0** | **0** | **0** | **0** | **0** | **0** | **0** | **0** | **0** | **0** | **11** | **0** | **0** | **0** | **0** | **0** | **0** | **0** | **0** | **0** | **0** | **0** | **0** | **0** | **0** | **0** | **0** |
| **Alytes obstetricans (Amphibia)** | **0** | **0** | **0** | **0** | **14** | **0** | **0** | **0** | **0** | **0** | **0** | **0** | **0** | **0** | **0** | **0** | **0** | **0** | **0** | **0** | **26** | **0** | **0** | **0** | **0** | **0** | **0** | **0** | **0** | **0** | **0** | **0** | **0** | **0** | **0** | **0** | **0** |
| **Pelobates cultripes (Amphibia)** | **0** | **0** | **0** | **0** | **0** | **0** | **0** | **0** | **0** | **0** | **14** | **0** | **0** | **0** | **0** | **0** | **0** | **0** | **0** | **0** | **0** | **0** | **0** | **0** | **0** | **0** | **0** | **0** | **0** | **0** | **0** | **0** | **0** | **0** | **0** | **0** | **0** |
| **Triturus marmoratus (Amphibia)** | **0** | **0** | **0** | **0** | **0** | **0** | **0** | **0** | **0** | **0** | **0** | **0** | **0** | **0** | **0** | **0** | **0** | **0** | **0** | **0** | **0** | **0** | **0** | **0** | **0** | **1411** | **1572** | **0** | **0** | **0** | **0** | **0** | **0** | **0** | **0** | **0** | **0** |
| **Anas platyrhynchos (Aves)** | **0** | **0** | **12** | **0** | **56** | **108** | **0** | **50** | **28** | **0** | **0** | **0** | **0** | **0** | **0** | **0** | **52** | **46** | **0** | **0** | **0** | **0** | **0** | **0** | **0** | **0** | **0** | **0** | **41** | **27** | **0** | **0** | **0** | **0** | **0** | **0** | **0** |
| **Corvus macrorhynchos (Aves)** | **0** | **0** | **0** | **0** | **0** | **0** | **0** | **0** | **0** | **0** | **11** | **0** | **0** | **0** | **0** | **0** | **0** | **0** | **0** | **0** | **0** | **0** | **0** | **0** | **0** | **0** | **0** | **0** | **0** | **0** | **0** | **0** | **0** | **0** | **0** | **0** | **0** |
| **Garrulus glandarius (Aves)** | **0** | **0** | **0** | **0** | **0** | **0** | **0** | **0** | **0** | **0** | **0** | **0** | **0** | **0** | **0** | **0** | **0** | **0** | **0** | **0** | **0** | **0** | **0** | **31** | **0** | **0** | **0** | **0** | **0** | **0** | **0** | **58** | **68** | **0** | **0** | **0** | **0** |
| **Riparia riparia (Aves)** | **0** | **0** | **0** | **0** | **0** | **0** | **0** | **0** | **0** | **0** | **0** | **0** | **0** | **0** | **0** | **0** | **0** | **0** | **0** | **0** | **0** | **0** | **0** | **301** | **0** | **0** | **0** | **0** | **0** | **0** | **0** | **0** | **0** | **0** | **0** | **0** | **0** |
| **Sturnus vulgaris (Aves)** | **0** | **0** | **0** | **0** | **0** | **0** | **0** | **0** | **0** | **0** | **0** | **0** | **0** | **0** | **0** | **0** | **0** | **0** | **0** | **0** | **0** | **0** | **0** | **0** | **0** | **0** | **12** | **0** | **0** | **0** | **0** | **0** | **0** | **0** | **0** | **0** | **0** |
| **Sylvia atricapilla (Aves)** | **0** | **29** | **0** | **0** | **0** | **0** | **0** | **121** | **51** | **0** | **0** | **0** | **0** | **0** | **0** | **0** | **0** | **0** | **0** | **12** | **25** | **0** | **0** | **0** | **0** | **42** | **99** | **0** | **0** | **0** | **0** | **19** | **13** | **0** | **0** | **0** | **0** |
| **Sylvia borin (Aves)** | **0** | **500** | **74** | **0** | **0** | **0** | **0** | **0** | **0** | **0** | **0** | **0** | **0** | **0** | **0** | **0** | **0** | **0** | **0** | **0** | **0** | **0** | **0** | **0** | **0** | **0** | **0** | **0** | **0** | **0** | **0** | **0** | **0** | **0** | **0** | **0** | **0** |
| **Erithacus rubecula (Aves)** | **0** | **0** | **0** | **0** | **11** | **14** | **0** | **826** | **455** | **0** | **0** | **0** | **0** | **0** | **0** | **0** | **0** | **0** | **0** | **0** | **0** | **0** | **0** | **0** | **0** | **0** | **36** | **0** | **0** | **0** | **0** | **17** | **0** | **0** | **13** | **0** | **0** |
| **Turdus merula (Aves)** | **0** | **0** | **0** | **0** | **384** | **313** | **0** | **0** | **0** | **0** | **28** | **0** | **0** | **0** | **0** | **0** | **0** | **0** | **0** | **0** | **0** | **0** | **0** | **0** | **0** | **42** | **30** | **0** | **0** | **0** | **0** | **13** | **0** | **0** | **0** | **0** | **0** |
| **Bos taurus (Mammalia)** | **0** | **126** | **516** | **0** | **2246** | **2427** | **0** | **83** | **78** | **553** | **121** | **248** | **0** | **1355** | **1456** | **0** | **93** | **30** | **0** | **998** | **721** | **0** | **675** | **117** | **0** | **0** | **32** | **0** | **42** | **0** | **0** | **1046** | **1338** | **0** | **233** | **165** | **0** |
| **Ovis aries (Mammalia)** | **0** | **14** | **0** | **0** | **0** | **0** | **0** | **0** | **0** | **0** | **302** | **709** | **0** | **0** | **0** | **0** | **11** | **0** | **0** | **0** | **0** | **0** | **0** | **0** | **0** | **0** | **0** | **0** | **0** | **0** | **0** | **71** | **138** | **0** | **0** | **0** | **0** |
| **Capreolus capreolus (Mammalia)** | **0** | **0** | **0** | **0** | **18** | **0** | **0** | **30** | **0** | **0** | **3306** | **4612** | **0** | **767** | **727** | **0** | **0** | **14** | **0** | **0** | **0** | **0** | **0** | **0** | **0** | **0** | **0** | **0** | **0** | **0** | **0** | **0** | **0** | **0** | **0** | **0** | **0** |
| **Cervus elaphus (Mammalia)** | **0** | **0** | **0** | **0** | **0** | **45** | **0** | **0** | **0** | **0** | **25** | **0** | **0** | **0** | **0** | **0** | **0** | **0** | **0** | **0** | **0** | **0** | **0** | **0** | **0** | **0** | **0** | **0** | **0** | **0** | **0** | **0** | **0** | **0** | **0** | **0** | **0** |
| **Sus scrofa (Mammalia)** | **0** | **40** | **74** | **0** | **13** | **19** | **0** | **0** | **147** | **0** | **0** | **19** | **0** | **15418** | **10783** | **0** | **100** | **214** | **0** | **24** | **261** | **0** | **0** | **0** | **0** | **62** | **0** | **0** | **0** | **0** | **0** | **660** | **397** | **0** | **36** | **0** | **0** |
| **Canis lupus (Mammalia)** | **0** | **0** | **0** | **0** | **0** | **25** | **0** | **0** | **0** | **0** | **0** | **0** | **0** | **0** | **0** | **56** | **0** | **0** | **0** | **0** | **0** | **0** | **0** | **0** | **0** | **0** | **0** | **0** | **0** | **104** | **0** | **0** | **17** | **0** | **0** | **0** | **0** |
| **Lutra lutra (Mammalia)** | **0** | **0** | **0** | **0** | **0** | **0** | **0** | **0** | **0** | **0** | **0** | **0** | **0** | **0** | **0** | **0** | **0** | **0** | **0** | **0** | **0** | **0** | **16** | **26** | **0** | **0** | **0** | **0** | **0** | **0** | **0** | **19** | **0** | **0** | **0** | **0** | **0** |
| **Mustela nivalis (Mammalia)** | **0** | **0** | **0** | **0** | **0** | **0** | **0** | **0** | **0** | **0** | **0** | **0** | **0** | **0** | **0** | **0** | **0** | **0** | **0** | **0** | **0** | **0** | **0** | **0** | **0** | **0** | **13** | **0** | **0** | **0** | **0** | **0** | **0** | **0** | **0** | **0** | **0** |
| **Pipistrellus pipistrellus (Mammalia)** | **0** | **0** | **29** | **0** | **0** | **0** | **0** | **0** | **0** | **0** | **0** | **0** | **0** | **0** | **0** | **0** | **0** | **0** | **0** | **0** | **0** | **0** | **0** | **0** | **0** | **0** | **0** | **0** | **0** | **0** | **0** | **0** | **0** | **0** | **0** | **0** | **0** |
| **Sorex araneus (Mammalia)** | **0** | **0** | **0** | **0** | **0** | **0** | **0** | **24** | **0** | **0** | **0** | **0** | **0** | **0** | **0** | **0** | **0** | **0** | **0** | **0** | **0** | **0** | **0** | **0** | **0** | **0** | **0** | **0** | **0** | **0** | **0** | **0** | **0** | **0** | **0** | **0** | **0** |
| **Oryctolagus cuniculus (Mammalia)** | **0** | **0** | **0** | **0** | **0** | **0** | **0** | **0** | **0** | **0** | **0** | **0** | **0** | **0** | **0** | **0** | **0** | **18** | **0** | **0** | **0** | **0** | **0** | **0** | **0** | **0** | **0** | **0** | **15** | **0** | **0** | **0** | **0** | **0** | **0** | **0** | **0** |
| **Equus caballus (Mammalia)** | **0** | **0** | **0** | **0** | **0** | **0** | **0** | **0** | **0** | **0** | **0** | **0** | **0** | **0** | **0** | **0** | **0** | **0** | **0** | **0** | **0** | **0** | **0** | **0** | **0** | **0** | **0** | **0** | **0** | **0** | **0** | **316** | **442** | **0** | **0** | **0** | **0** |
| **Homo sapiens (Mammalia)** | **31** | **153** | **50** | **20** | **253** | **176** | **0** | **55** | **78** | **1275** | **101** | **55** | **0** | **0** | **0** | **0** | **67** | **22** | **107** | **44** | **178** | **1842** | **24** | **2055** | **87** | **0** | **21** | **31448** | **12** | **14** | **0** | **634** | **596** | **40** | **71** | **26** | **0** |
| **Arvicola amphibius (Mammalia)** | **0** | **0** | **0** | **0** | **37** | **83** | **0** | **0** | **0** | **0** | **0** | **0** | **0** | **0** | **0** | **0** | **0** | **0** | **0** | **0** | **0** | **0** | **0** | **0** | **0** | **0** | **0** | **0** | **0** | **0** | **0** | **0** | **0** | **0** | **0** | **0** | **0** |
| **Microtus arvalis (Mammalia)** | **0** | **0** | **0** | **0** | **0** | **0** | **0** | **338** | **631** | **0** | **0** | **0** | **0** | **0** | **0** | **0** | **0** | **0** | **0** | **0** | **0** | **0** | **0** | **0** | **0** | **27** | **0** | **0** | **0** | **0** | **0** | **0** | **18** | **0** | **0** | **0** | **0** |
| **Apodemus sylvaticus (Mammalia)** | **0** | **0** | **0** | **0** | **0** | **0** | **0** | **0** | **16** | **0** | **0** | **0** | **0** | **0** | **0** | **0** | **0** | **0** | **0** | **0** | **0** | **0** | **0** | **0** | **0** | **0** | **0** | **0** | **0** | **0** | **0** | **16** | **0** | **0** | **0** | **0** | **0** |
| **Emys orbicularis (Testudines)** | **0** | **0** | **0** | **0** | **0** | **0** | **0** | **0** | **0** | **0** | **0** | **0** | **0** | **0** | **0** | **0** | **0** | **0** | **0** | **0** | **0** | **0** | **39** | **0** | **0** | **0** | **0** | **0** | **0** | **0** | **0** | **0** | **0** | **0** | **0** | **0** | **0** |

| **ID M2**  **Species** | **AT5166_BL** | **AT5166_L1** | **AT5166_L2** | **AT5167_BL** | **AT5167_L1** | **AT5167_L2** | **AT5168_BL** | **AT5168_L1** | **AT5168_L2** | **AT5169_BL** | **AT5169_L1** | **AT5169_L2** | **ATBPCRA** |
| --- | --- | --- | --- | --- | --- | --- | --- | --- | --- | --- | --- | --- | --- |
| **Triturus marmoratus (Amphibia)** | 0 | 0 | 11 | 0 | 0 | 0 | 0 | 0 | 0 | 0 | 0 | 0 | 0 |
| **Anas crecca (Aves)** | 0 | 0 | 0 | 0 | 0 | 11 | 0 | 0 | 0 | 0 | 0 | 0 | 0 |
| **Anas platyrhynchos (Aves)** | 0 | 59 | 156 | 0 | 55 | 0 | 0 | 40 | 36 | 0 | 74 | 43 | 0 |
| **Aegithalos glaucogularis (Aves)** | 0 | 0 | 0 | 0 | 0 | 0 | 0 | 0 | 0 | 0 | 15 | 0 | 0 |
| **Pica pica (Aves)** | 0 | 0 | 0 | 0 | 11 | 0 | 0 | 0 | 0 | 0 | 0 | 0 | 0 |
| **Phylloscopus canariensis (Aves)** | 0 | 0 | 0 | 0 | 11 | 0 | 0 | 18 | 0 | 0 | 0 | 0 | 0 |
| **Sylvia atricapilla (Aves)** | 0 | 0 | 0 | 0 | 0 | 0 | 0 | 11 | 93 | 0 | 0 | 0 | 0 |
| **Erithacus rubecula (Aves)** | 0 | 0 | 0 | 0 | 0 | 0 | 0 | 0 | 0 | 0 | 13 | 0 | 0 |
| **Turdus kessleri (Aves)** | 0 | 0 | 0 | 0 | 0 | 0 | 0 | 37 | 0 | 0 | 0 | 0 | 0 |
| **Turdus merula (Aves)** | 0 | 0 | 0 | 0 | 0 | 0 | 0 | 50 | 62 | 0 | 0 | 0 | 0 |
| **Turdus philomelos (Aves)** | 0 | 0 | 0 | 0 | 0 | 0 | 0 | 53 | 0 | 0 | 0 | 0 | 0 |
| **Bos taurus (Mammalia)** | 0 | 196 | 399 | 383 | 277 | 258 | 0 | 51 | 11 | 0 | 6983 | 5992 | 0 |
| **Ovis aries (Mammalia)** | 0 | 0 | 0 | 0 | 0 | 15 | 0 | 0 | 0 | 0 | 0 | 14 | 0 |
| **Capreolus capreolus (Mammalia)** | 0 | 0 | 24 | 0 | 0 | 0 | 0 | 0 | 0 | 0 | 0 | 0 | 0 |
| **Cervus elaphus (Mammalia)** | 0 | 0 | 0 | 0 | 0 | 0 | 0 | 0 | 0 | 0 | 0 | 21 | 0 |
| **Sus scrofa (Mammalia)** | 0 | 21 | 77 | 0 | 128 | 24 | 0 | 106 | 47 | 0 | 45 | 136 | 0 |
| **Canis lupus (Mammalia)** | 0 | 0 | 0 | 0 | 17 | 21 | 0 | 0 | 0 | 0 | 0 | 0 | 0 |
| **Vulpes vulpes (Mammalia)** | 0 | 0 | 0 | 0 | 0 | 0 | 0 | 0 | 12 | 0 | 0 | 0 | 0 |
| **Lutra lutra (Mammalia)** | 0 | 0 | 13 | 0 | 0 | 0 | 0 | 0 | 0 | 0 | 22 | 0 | 0 |
| **Homo sapiens (Mammalia)** | 71 | 36 | 40 | 249 | 189 | 183 | 1064 | 53 | 0 | 0 | 15 | 1858 | 17 |
| **Microtus arvalis (Mammalia)** | 0 | 0 | 0 | 0 | 0 | 0 | 0 | 0 | 0 | 0 | 0 | 11 | 0 |
| **Microtus lavernedii (Mammalia)** | 0 | 0 | 0 | 0 | 14 | 0 | 0 | 0 | 0 | 0 | 0 | 0 | 0 |
| **Myodes glareolus (Mammalia)** | 0 | 0 | 0 | 0 | 0 | 0 | 0 | 0 | 0 | 0 | 15 | 0 | 0 |
| **Apodemus sylvaticus (Mammalia)** | 0 | 0 | 0 | 0 | 0 | 0 | 0 | 0 | 0 | 0 | 101 | 116 | 0 |
