# Supplementary material for: Environmental DNA metabarcoding of water samples as a tool for monitoring Iberian freshwater fish composition
Source: PLoS One. 2023 Oct 30;18(10):e0283088. doi: 10.1371/journal.pone.0283088 (PMC10615313; doi:10.1371/journal.pone.0283088)
Supplement: Attachment — Submitted filename: eDNA review detailed comments.docx [file pone.0283088.s002.docx]

Specific comments organized by order of the manuscript are listed below.

Abstract:

Line 26: What do the authors mean by primary freshwater fish? Does this mean the most common species? Unsure if “primary” is the correct word

Line 33: Be clear that the population-level differences in 12S gene for A. Arcasii was detected from tissue samples and not from eDNA. The way it is written now is misleading.

Line 37: “Need to generate a local database” – the authors should be clear what kind of database this is referring to. I’m assuming a database for 12S gene sequences for known local fish species?

Line 39: Final sentence in the abstract. Be explicit if the study showed eDNA can be an early detection method for invasive species, or if this is referring to eDNA studies in general

I’d like to see a brief mention in the abstract of how this study impacts knowledge/conservation/ management of Iberian peninsula fish populations specifically.

Introduction:

Lines 51-59: Good points about the limitations of traditional sampling methods but I think the authors should also explicitly mention problems with gear bias and how that might lead to underestimating species composition and diversity (for example mesh size of nets, net location in water column or habitat)

Lines 68-70: This last sentence could be expanded; what do the authors mean by each of these problems with eDNA? Explain and give examples of each. It’s probably also worth mentioning problems with contamination, false negatives and false positives and specifically how each of those could occur.

Lines 71-85: This paragraph mostly seems like it would fit better in the discussion when mentioning any potential shortcomings of the study. The authors make a good point in mentioning other mitochondrial markers and limitations/benefits of each. I am also curious if the authors considered using more than one gene as other eDNA studies have done to reduce the potential shortcomings of using a single gene? I’d like to see some discussion about this, if not here then in the discussion.

Line 82: “The reference database for 12S” – be clear about which database this refers to. GenBank?

Lines 86-92: If the fish species in this area lack data for the 12S gene why was it used instead of the “markers of choice”? If the point of this study was to show that the 12S gene can be used to detect native Iberian species the authors should be explicit about that and state that as a clear objective in the introduction. Otherwise, why weren’t the other genes used?

Line 87: “As with many other of these types of studies” – An example of vague wording. Be clear about what type of study this is referring to. eDNA studies? Phylogenetic studies?

Lines 93-94: This paragraph should start with the description of the Iberian peninsula and then introduce the description of the Duero basin (start broad and zoom in).

Lines 94-97: Before introducing examples of threatened species, I would like to see some broader regional context and discussion of some of the problems within the basin that are leading to declines in certain fish populations. This is mentioned at the end of the paragraph but should be introduced sooner for readers who are not familiar with the region or the specific species mentioned earlier.

Lines 98- 100: The authors mention the distribution and population genetics of “several species” – be specific about which species these are and give examples. Give some context maybe earlier in the paragraph – approximately how many species exist in the peninsula or the basin? How many native vs introduced? How many is “several”? Are many species declining or just a few?

Lines 102- 104: “..64% are included in one of the three most critical categories..” – what does this mean exactly? What are the three categories? What is the “one” category this refers to? Be clear and specific.

Lines 106-107: “For several of the species”, again be specific about what “several” means and how that compares to other species, maybe give some examples.

Line 108: “High percentage” – percentage of what? Perhaps this should just refer to the number of different species in the basin rather a percentage of something.

Lines 108-110: This sentence could be broken up and clarified. Are the authors really concerned about the strong threat to exotic species?

Lines 110-114: The objective(s) should be very clear and specific and relate to what the authors did in the study. Based on other sections of the paper, it appears one of the objectives was to compare eDNA metabarcoding to electrofishing samples. Be clear if this was the case. Based on earlier paragraphs and discussion of other mitochondrial markers, it seemed that another objective was to assess if 12S could be used to detect different fish species in this region. Was another objective to assess genetic differentiation or identify population-level differences for A. arcassii based on known tissue samples? If it wasn’t, why was that included in the study?

Materials and Methods

Line 121: I’m not sure if “locality” is the best choice of word although I might be biased in my USA vocabulary. Something like “sampling site” or sampling location” seems more fitting for scientific studies

Line 123: What were the ichthyological samplings? What methods were used?

Line 124: “The representative sample of all the fish..” Does this mean a representative sample of all the fish species present? Or some other metric? Be specific

Lines 126-127: How much time between eDNA sampling and electrofishing? This could be very important

Lines 128-130: The authors mention a reservoir but readers who are unfamiliar with this region don’t know what reservoir this is referring to. Perhaps some more background on the sample sites, or a description of in table 1 about what kind of system each site is (reservoir/lake/large river/small river/stream) would be helpful

Figure 1: This map is helpful, but coordinates should be labeled with units.

Table 1: Caption for the table should give descriptions of the columns. At this point the reader does not know what ID_M1 and ID_M2 mean. It would also be helpful to include some more site descriptions for people not familiar with this region, like I mentioned previously is it a lake/reservoir/river/stream? Is it in the headwaters or lower in the basin? What kind of land use is dominant in the area? Urban/farmland/forestland? Highly impacted by humans or more “natural”? I would like to have some context to these sites. It would also be helpful to have the sampling dates of eDNA sample collection and traditional sampling at each site

Lines 139-140: Please explain why only four sites were resampled in October and why those sites were chosen over others.

Lines 139-150: The authors should be more specific and detailed about the steps of collecting and filtering water samples and also build this paragraph in the order of the collection and filtering process so the reader can easily follow along. For example, filtering is currently mentioned before the description of water collection. More details are also necessary. How were water samples collected? From a boat or from the side of a river? Was the collector wearing gloves? Which equipment specifically was disinfected? What does filtering in situ mean? On a boat? On the side of a river? In a car? What was used to filter the water? Someone should be able to replicate exactly the sampling process based on the methods and right now a lot of the necessary detail isn’t included.

Lines 147-148: Why were filtered samples re-filtered through a 0.2 um filter?

Lines 152-155: Be clear before describing this step that this is the exception to the protocol previously mentioned.

Lines 159-160: Who were the relevant authorities?

Lines 158-166: Need more detail and clarification on how sample sites were selected and what kind of electrofishing was used. Where was the eDNA sampling site in relation to the electrofishing site at each location? The authors mention transects were sampled twice – how much time between the two surveys? What specific mesohabitats were surveyed? Wouldn’t there have been issues accessing some habitats while electrofishing? Again it would be useful to describe the whole process sequentially and in detail so the reader could easily replicate what the authors did.

Lines 168-173: I think the authors need to be more specific on how these inventories were constructed. Did they create a list of all species observed from their electrofishing surveys and then add data from the mentioned previous projects? Was angler data only included in the Juarros de Voltoya site and not any others? The last sentence also mentions “data gathered in previous years”, the authors should be specific on what exactly this data is and what years specifically it was from.

Line 175-176: Do the 48 samples include the 4 resampled sites? The wording is somewhat confusing

Line 202: What is the unpublished data?

Line 202-203: Which was the one species from Morocco?

Line 215: Is 149 referring to the combined number of unique sequences across the 26 species previously listed? It would be helpful to know the range of number of sequences by species, for example did some species only have 1 sequence in GenBank while others had 40?

Lines 215-218: I’d like to see more detail about these additional sequences. Were the additional 19 species totally different from the previous 26? How much overlap was there? Again what was the range of the number of sequences per species? How old were the samples, how were they stored, and was there any concern for DNA degradation?

Line 231: How were sequences manually examined? Some more detail here would be useful.

Line 232-233: What was this already existing database? Is this different from the database the authors built? How were the sequences linked to this database? This description is lacking detail

Lines 235-238: The authors should give some context as to why this analysis was performed. Was an objective of the study to assess genetic differentiation across populations? What samples was this assessment based on? Known tissue samples or eDNA samples? If this assessment was based on tissue samples the authors should be clear and describe if they came from electrofishing samples or archived samples or other. This should either be explicitly included as an objective earlier in the manuscript or not included as it is not relevant to the eDNA assessment.

Lines 252-257: The wording here is somewhat confusing. Were unassigned OTUs totally removed or were they mapped to GenBank to see if they matched to any species that might not have been in the authors’ database? The authors state they only kept reads that 100% aligned to known sequences from GenBank – what exactly does that percentage mean if they also set a 97% similarity threshold?

Did the authors consider doing any kind of statistical comparison in the number of species detected with each method and if it was significantly different? Or to test a difference in species between seasons for the 4 sites sampled twice? This is necessary to make any claims in differences in the Results and Discussion.

Results

The order of the sections within the results should mirror the methods and put the most important parts of the study first

Line 260-262: It would be more useful to present this information in terms of ranges of different sequences per species and number of haplotypes per species. If this is referring to the results from known tissue samples the authors should be clear on that; the way it is presented now makes it sound like these results are referring to eDNA results which is confusing. Be clear on where the “new” sequences are coming from – electrofishing or archived samples or both?

Lines 262-270: See above comments on whether this analysis is relevant to the stated objectives and should even be included.

Table 2: Why were some groups of species assessed for interspecific similarity and not others? Is this analysis meaningful to the main objectives of the study?

Lines 283-291: So when the authors blasted their unassigned reads they didn’t match to any fish specifically? Only to Actinopterygii as a broad group? Were there any matches to smaller groups of fishes? It seems odd that matches would jump from species level all the way up to Order I’d also like to see some discussion in this paragraph of the range of reads in the field blanks and lab blanks.

Line 289: “The vast majority of reads.” Does this refer to non-fish reads? Or the reads after the authors excluded Actinopterygii? The language is somewhat confusing

Figure 2: Having the different y axes labelled would be helpful. I’m also not sure if using lines is the best way to present this data as lines typically show trends/changes, where here we are looking at one value (reads) per independent sample, therefore the lines are somewhat misleading. The caption should also define the components of the plot (site name, site number, BL, FL, L2, etc). Also does this plot show the reads for the sites that were sampled in October? Are reads from the two sampling times combined? The authors should be explicit if they are or otherwise. The sites sampled twice should also have some sort of symbol or asterisk to demonstrate which they are.

Figure 3: It would be helpful to label M1 and M2 in the plot or just label them “summer” and “autumn”. I think the 99% and 97% label are somewhat confusing – either they should be called something else or defined clearly in the caption. The authors should clarify what they mean by “to the species level” in the caption. So is everything in green something that was assigned all the way down to species level or does it include identified genera or families too? Are we potentially missing out on data if reads were not assigned all the way down to species? It also appears that there is a typo in panel A – should the blue be labeled Actinopterygii? Why is this bold and underlined? Caption describing panel B should be clear that these are reads that were originally unassigned to the reference database and that these are the results after blasting to GenBank. Also think back to the main objectives and if this information on other orders is even meaningful or useful for the study

Line 305: The authors should be explain this “matrix”; there was no previous mention of it.

Lines 307-310: It would be helpful to show any assignments to genus in the figures (specifically Figure 4) so the reader can see which genera were detected in which locations. Otherwise, we might be missing data in our interpretation of the results

Line 311: Are the 3 genera different from the 31 species or do they overlap?

Lines 313-320: I would move this entire discussion of the marine species to a separate paragraph and keep the flow of the paragraph logical (introduce exceptions then immediately describe them and give examples)

Lines 323-325: Do the authors think that the reads that matched to L. rifensis were actually from the closely related L. bocagei? If this is the case it should explicitly explained. Otherwise, is it totally impossible that L. rifensis could be in the basin? This might need to be explained for readers who aren’t familiar with this region and species. Or if the authors are unsure, couldn’t this assignment be to common genus?

Line 325: Again, is it totally impossible that something in the Leuciscus genus could be in the basin just because it hadn’t been sampled before? How often did this genus appear in the samples? I am nervous that the authors may be losing meaningful results by discarding certain genera.

Lines 329-332: If the authors decided to keep P. polylepis that should be explicitly stated.

Line 333: “A total of 22 species” – does this include genera that were not able to be classified down to species? If so, perhaps calling them “taxonomic units” or “taxa” would be better. If the authors are excluding any matches to genera/family, are they not losing potentially valuable data?

Lines 334-342: I think it would be logical to move this paragraph earlier in the results when first discussing the number of reads and matches coming out of the metabarcoding pipeline. For the species that showed up in the field blanks – are they present in high numbers in those rivers i.e., does contamination from that species make sense? Is it possible they could have been contaminated in the lab too? (Perhaps this is more discussion material)

Lines 343-345: This sentence is somewhat confusing and vague; each table and figure should be referred to clearly.

Line 346: “Considering all cases” – what does this mean? The authors should avoid vague language and be explicit in what exactly they are referring to.

Line 347-348: What do the authors mean by species detected by the traditional method were not in the present study? This language is confusing and makes it sound like there were no species detected in the authors’ electrofishing sampling

Figure 4: I think there is a better way to present these results. Perhaps have all the sites on one axis and all potential species on the other and create a kind of heatmap where shading of color represents number of reads (or percentage) for a specific species at a specific site. This would be easier for the reader to visualize species across sites and see which species are common, rarer, or might only be present in one or two locations. In the caption, define what percentage the authors are referring to – number of reads?

Figure 5: I think it would be best to just label each subplot rather than making the reader decipher the caption. Also the authors should be clear if the traditional methods results in the first panel include past samples or just the current electrofishing results. It might even be worth separating out past traditional sampling to the current study. I think the wording in the caption could also be more clear and list the panel letter before the description (e.g., “Comparison of the number of species detected for A) eDNA and traditional sampling (TS), B)….”)

Table 3: The caption refers to numbers in ED columns as mean relative abundance – does this mean percentage of reads in the sample aka the same values in Figure 4? The wording should stay consistent, or perhaps not be presented twice in a table and also in a figure. There is a lot for the reader to process and decipher in this table and I almost wonder if it should be broken up into more than one thing. If the percentage of reads is also presented in Figure 4 perhaps this table would be less overwhelming if it just included presence/absence of species in each separate method/sampling time, or perhaps separate out the comparison of past to current traditional sampling in a different table or figure.

Lines 374-382: This entire paragraph is extremely difficult to follow, especially the second sentence. The authors should reflect on the main point they’re trying to make and explain it in a clear and simple manner. Instead of presenting percentages or variations in number of species in one or two liters, it would be much easier for the reader to comprehend if the authors just presented counts (e.g., the average number of species detected in each sample liter was 4.2 and ranged from 2 to 11). Also consider performing some kind of statistical comparison.

Line 378: What is a “station”? Is this the same thing as a sampling site or “locality? – wording should stay consistent throughout.

Lines 383-389: I’m not sure how this section ties into the objectives. If a goal of the study was to compare native and introduced species detected with eDNA, that should be stated previously and described in the methods.

Line 384: I am confused about the mention of the two species of undetermined origin. I do not remember seeing anything about this previously and I’m not sure what it means. Please explain this more fully either here or in the methods.

Line 338-389: This sentence is very confusing and the authors should be specific in which rivers they’re referring to. Currently this makes it sound like no native species have been reported in some of the rivers which doesn’t make sense.

Figure 6: Coordinates should be labeled with unit

Discussion

Sections of the discussion should reflect the objectives and main study goals

Lines 402-404: Does this mean C. vettonica was not included in the reference database or does it mean it was not detected in any of the eDNA samples? Be clear. If this result was not previously mentioned in the Results section (which I don’t remember it being stated) it should first be presented there. Remember, no new results should appear in the discussion.

Lines 404-405: It would be useful to know what kinds of organisms these previous studies were on and how different the authors’ study is in comparison

Line 406: “a large number of..” is this referring to number of species in the reference database? In the eDNA results? Or both? One could argue most eDNA metabarcoding studies are assessing a “large number” of species.

Lines 409-410: Be clear that this refers generally to previous studies. Also are all of these studies on fish or aquatic organisms?

Line 411: “with fewer than 100 species” why is that relevant? If this is important it should be mentioned in the Introduction but I would argue that this point isn’t important to the authors’ study.

Lines 411-413: It is difficult to make this claim without a statistical analysis of some sort. See previous comment in Methods

Line 413: Be clear what the 83.33% refers to.

Lines 413-414: “However,..” this sentence could be worded more clearly.

Lines 420-421: How far away are the locations upstream where the fish were recorded? Would this make sense with the eDNA results? Is it 1 km away? 100? Why wouldn’t the authors expect to detect this species in their sampling location? Remember to provide context for readers unfamiliar with these species and rivers.

Lines 430-431: Very interesting about the marine species likely showing up from human consumption and waste. Were the sites that these species were detected downstream from urban areas or wastewater treatment plants? Are there other previous eDNA studies that have shown this phenomenon? It would be useful to compare and site them here.

Lines 431-434: Is there a citation to support this claim? In theory even if DNA is present in low concentrations, it should still be able to be amplified and detected, correct?

Line 435: Be clear what this 5.38% percent refers to.

Line 437: “under this approach” – avoid vague terminology and be specific

Lines 437-439: I’d like to see some more discussion and examples of these factors. For example, how would life cycle affect eDNA presence?

Lines 443-444: How far away from the reservoir did electrofishing take place? Could the discrepancy in species detected for Juarros del Voltoya also be explained by differences in fish communities of a lake habitat vs a river habitat? This might be good for the authors to discuss here

Lines 444-448: These points seem somewhat contradictory. If eDNA rapidly declines in lotic systems, wouldn’t eDNA sampled in lentic systems be more reliable? But this does not seem to be what the authors are saying. What do the authors mean by “spatial structure” of eDNA? They should be specific about what the degree of water mixing by seasons might do to eDNA and how that might affect their study.

Lines 450-451: Is this referring to previous eDNA studies for fish? The authors should be specific

Line 451: The authors should be explicit about what “different optimal methodologies” means and what kinds of studies that refers to. Again, is this fish-specific?

Line 455-456: I would also like to see some discussion on the potential problem of sampling at just one location in a large river system, and how specific sampling location might also affect results (i.e. collecting river samples from one location at a river bank, less than 1 m into the river in a river that might be 50 m across). As mentioned previously it would be useful to know the characteristics and water body size of the sampling locations. A single sample site might make sense in a small creek, but I would think in a large river you’d likely be missing a good amount of DNA present in the system, and I’d like the authors to reflect on this.

Line 456-457: Statistically significantly different? Otherwise, the authors cannot make this claim

Line 458-459: I’d like to see an explanation of how these fish species move and migrate throughout the year in this basin, or perhaps an example species from this study that would explain the difference in detection between summer and autumn. Otherwise, couldn’t the amplification of different species from one sampling time to the next be completely random? How do the authors know it’s not?

Line 461: “reads” – be clear this refers to “read counts” or “quantity of reads”

Lines 461, 463: This sentence is completely contradictory, perhaps change the wording

Lines 467-468: These species weren’t detected in traditional sampling at all? How many species was this? How often were they only detected in eDNA vs also in traditional sampling? It would be useful to know the actual numbers, and if this isn’t presented in the Results it should be.

Line 470-471: If the authors did observe a correlation between abundance and eDNA read count this should be listed as an objective and described in the Methods and the Results. Was this correlation observed for all species? Which ones? Also, some sort of statistical test on this relationship is necessary to make any conclusions.

Line 471: Explain some of these biotic and abiotic factors and give examples.

Lines 472-473: What kind of studies are the authors suggesting? They should be more specific. Water quality studies? Further fisheries sampling? Habitat surveys? Geomorphology?

Lines 475-476: What about the instances that the 12S gene couldn’t classify down to species?

Line 482-484: The authors acknowledge the high variability within A. arcasii, but is there a potential for high interpopulation genetic variability within other species as well? What is the potential for misassignment of other species because of potential variation, even if it has not yet been documented for other species in this region? It is also well documented for some closely related species to have up to 100% similarity for a specific gene (including 12S), so how did the authors account for this possibility and ensure they were totally confident in species assignments? I’d like to see some discussion of this.

Line 489: be specific about what archived samples this refers to; archived water samples? eDNA filters? Tissue samples? How long would these samples be good enough quality to reanalyze later?

Lines 489-491: Using an additional gene for broader taxonomic coverage would also be beneficial

Lines 493-500: I am confused how changes of two other rivers might have affected fish composition in the Adaja; please describe how these things connect and remember that readers likely aren’t familiar with all of these rivers so please provide some context.

Line 495: Please define the Tajo-Segura transfer

Line 496 and 500: Please define stream captures and stream piracy.

Line 498: Please describe what you mean by movement of ichthyofauna; temporary? Permanent? Migration?

Line 500-501: Is this species known to be used for bait? Does that hypothesis make sense?

Line 503: “In some rivers..” this should be the beginning of a new paragraph

Line 503-513: I agree that the potential for hybridization can confound eDNA results, but how can the authors be sure this is happening? I think it is a stretch to claim that “it is likely that hybridization zones exist for these species in these rivers”. It seems very possible that one of those species truly exists in that specific river and just wasn’t sampled by electrofishing. Or if those species are closely related enough to hybridize, how can the authors even be sure any of their species assignments are correct?

Lines 518-520: The authors seem to be aware of the potential misassignment of species due to high similarity to other species, and based on this example of L. aspius and A. alburnus, the reader can’t help but wonder how much of these and other species detections can be trusted? I think the authors may need to go back and reassess some of their assignments, and if there are high percentage matches to multiple species, choose to be conservative and assign back to a common genus or family.

Line 528-530: Please give examples of the invasive species detected. Are they all expanding? What are the authors using to compare this to and make this conclusion?

I’m not sure if the concluding paragraph should solely discuss invasive species. Was assessing the presence of invasive species a main goal? The concluding paragraph should wrap up all the study objectives, summarize any limitations, and touch on specific management, conservation, or suggested future research.

General language issues:

- Choice of wording: I may be biased based on my country of origin (US) but I have never seen “autochthonous” and “allochthonous” used to refer to native vs introduced species. I find it distracting while reading but if those terms are commonly used and understood in other regions then ignore me.

- Some choice of wording is unclear, vague, or misleading. Avoid words like “some” and “several” which appear often – descriptions should be as specific as possible.

- Many sentences could be trimmed up and simplified; some are quite wordy and hard to follow due to confusing sentence structure or excess words that are unnecessary. For example line 158: “The traditional sampling approach of electrofishing was carried out..” could be “Electrofishing occurred..”

- This may be a personal preference, but the excessive use of passive voice is distracting

- Many phrases or word choices are informal or not suitable for scientific writing. Example line 511 “both their DNAs…” should be something like “the DNA of both species...”
